# A Unique Case of Muscle-Invasive Metastatic Breast Cancer Mimicking Myositis

**DOI:** 10.1155/2017/2648296

**Published:** 2017-06-28

**Authors:** Janelle Gyorffy, Samuel M. Philbrick, Adrian R. Bersabe, Richard J. Upton, Derek A. Mathis, Austin Peters, Alexander Brown

**Affiliations:** ^1^Department of Internal Medicine, SAUSHEC, San Antonio, TX, USA; ^2^Department of Hematology Oncology, SAUSHEC, San Antonio, TX, USA; ^3^Department of Pathology, SAUSHEC, San Antonio, TX, USA; ^4^Department of Radiology, SAUSHEC, San Antonio, TX, USA

## Abstract

Breast cancer rarely metastasizes to the muscles, and it is even more unusual for this phenomenon to result in airway compromise. We present a unique case of an 84-year-old female who presented with neck swelling and upper airway obstruction due to metastatic breast cancer invading the sternocleidomastoid muscles. After establishing the diagnosis and discussing possible treatment options, the patient elected for antiestrogen therapy, palliative tracheostomy, radiation therapy, and hospice services.

## 1. Introduction

Breast cancer is the most commonly diagnosed cancer in women. The incidence of breast cancer increases with age, and a woman's lifetime risk is approximately 12.5%. While breast cancer is diagnosed at stage 1 in approximately half of non-Hispanic white women, diagnosis of metastatic disease as the initial presentation is seen in 6% of patients [1]. Breast cancer typically metastasizes to lung, liver, and bone [2]. Involvement of skeletal muscle is rare, but when it occurs, it usually presents with contiguous involvement of a soft tissue lesion and may appear clinically as a lump or mass. It may also occur in the setting of extensive bone involvement or in the presence of widely disseminated disease [3, 4]. Extranodal head and neck metastases are also uncommon with only a few case reports and case series documenting involvement of the thyroid, mandible, subcutaneous tissues, or pharynx [5–11]. Thus, metastases to muscles of the neck are almost unheard of, with only one such case documented in medical literature [12]. We report the exceedingly unusual case of an 84-year-old African-American female with neck swelling and airway obstruction due to metastatic breast cancer invading the musculature of the head and neck.

## 2. Case Report

An 84-year-old African-American female with no known history of malignancy initially presented at a local Emergency Department (ED) with complaint of dyspnea for several months. Patient was diagnosed with asthma and given an albuterol inhaler. Five months later, she presented to our ED with progressive neck swelling and dysphagia. Urgent evaluation with Otolaryngology (ENT) was arranged as she was noted to have difficulty controlling her secretions. During ENT evaluation, she acutely developed respiratory failure requiring emergent fiber-optic nasotracheal intubation and transfer to the medical intensive care unit (MICU).

**Figure 1:**
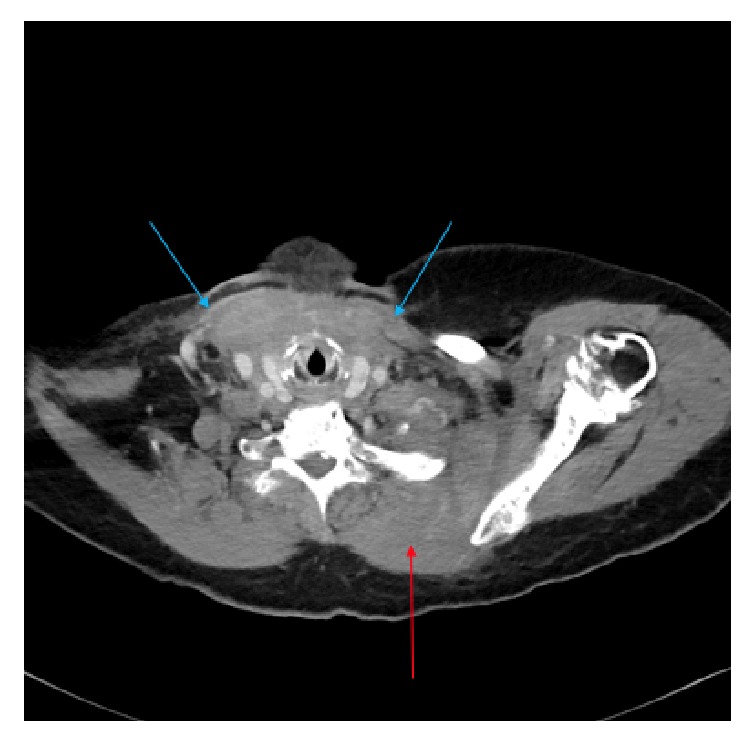
Significant thickening of the sternocleidomastoid muscles (blue arrows). Soft tissue density obliterating numerous fat planes extending into the mediastinum and completely encasing the major vessels and left paraspinal musculature including the left trapezius muscle (red arrow). The very few cases of breast cancer metastatic to the soft tissues predominantly describe focal or mass-like involvement. This diffuse, infiltrating appearance is highly unusual and most characteristic of severe infection/inflammation such as myositis or necrotizing fasciitis.

A CT of the neck, chest, abdomen, and pelvis was remarkable for extensive inflammatory stranding of multiple muscular and fascial planes in the neck, mediastinum, and anterior chest. There was also moderate narrowing of the supraglottic airway (Figure 1). There was no evidence of other evidence of distant metastases. The CT did not reveal any bony lesions. A PET-CT and bone scan were unable to be performed given patient's unstable condition in the MICU.

Erythrocyte sedimentation rate (ESR) was within normal limits at 26 mm/hr, and C-reactive protein (CRP) was undetectable. Lactate dehydrogenase (LDH) was within the labs reference range at 155 U/L, and aspartate aminotransferase (AST) was normal at 26 U/L. Creatinine kinase (CK) was mildly elevated at 239 IU/L. Evaluation for an autoimmune etiology was negative, and the patient had no clinical improvement with high dose steroids.

**Figure 2:**
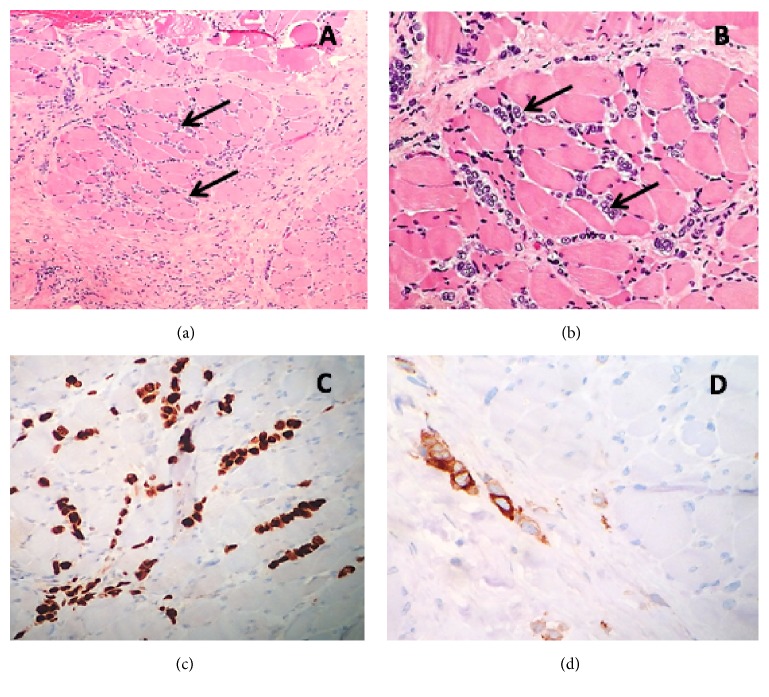
(a) Malignant cells (arrows) infiltrating through skeletal muscle and fibroconnective tissue (hematoxylin-eosin, original magnification ×100). (b) A higher power view shows malignant cells infiltrating singly or in small clusters through muscle and fibroconnective tissue. No tubule formation is identified, which is typical of invasive lobular breast carcinoma (hematoxylin-eosin, original magnification ×200). (c) Malignant cells alone and in single file strongly immunoreactive for GATA-3, a luminal marker for breast epithelium (original magnification ×200). (d) Malignant cells showing strong immunoreactivity for mammaglobin, another marker specific for breast carcinoma (original magnification ×400).

A biopsy of the left sternocleidomastoid (SCM) was obtained which revealed infiltrating carcinoma. No associated inflammatory infiltrate or necrosis was identified. The tissue was strongly positive for estrogen receptor (ER), progesterone receptor (PR), pancytokeratin (Lu-5), high molecular weight cytokeratin (K903), GATA-3, and mammaglobin. Cells were mildly to moderately positive for GCDFP-15 and E-cadherin, and negative for HER-2/neu, S-100, TTF-1, CD34, CD30, and PAX-8 (Figure 2). Staining with mammaglobin showed strong positivity within malignant cells. These immunohistochemical findings were most consistent with a breast primary malignancy. Biopsies from vocal cord, arytenoid, epiglottis, and uvula biopsies demonstrated no malignant cells.

The patient had no palpable breast masses, and no axillary, supraclavicular, or cervical lymphadenopathy was clinically appreciable. A bilateral breast ultrasound showed a hypoechoic 1.7 × 1.0 × 1.5 cm mass in the left breast with increased vascularity highly suggestive of malignancy (BI-RADS 5) and a 1.1 × 1.1 × 0.5 cm mass in the right breast without increased vascularity also suspicious for malignancy (BI-RADS 4). There were no pathologic axillary lymph nodes appreciated on imaging. Serum tumor markers were remarkable for elevating CA 15-3 to 299 U/mL and CA 27.29 to 238 U/mL. CEA was elevated just above the reference range at 3.5 ng/L.

The diagnosis of metastatic breast cancer was discussed with the patient and her family. Given her critical illness and patient's desire for a less aggressive palliative approach, the patient elected for antiestrogen therapy with Fulvestrant over a trial of single-agent chemotherapy. She also agreed to palliative tracheostomy and radiotherapy (XRT) to expedite ventilator weaning. She underwent the four fractions of palliative XRT at 370 cGy for a total dose of 1480 cGY. She was successfully weaned off of ventilator support and transitioned to home hospice. The patient declined further treatment. She passed away approximately one month later after the definitive diagnosis of metastatic breast cancer was made.

## 3. Discussion

Breast cancer most frequently metastasizes to bone (70%), lung (66%), and liver (61%) but may involve other organs [2]. Malignant airway obstruction is more commonly found in patients with advanced local lung cancer but may rarely result from metastasis from cancers of the thyroid, breast, colon, melanoma, or kidneys [13]. This case demonstrates a rare instance of metastases to the SCM muscles along with the unusual presentation of airway obstruction due to local skeletal muscle invasion manifesting as myositis.

The patient's myositis caused by infiltrating carcinoma presented in a manner distinct from dermatomyositis (DM) and polymyositis (PM), idiopathic inflammatory myopathies that typically present with proximal muscle weakness and muscle inflammation. The differential diagnoses for DM and PM are broad and include sarcoidosis, amyloidosis, drug-induced myositis, and other infectious, familial, and autoimmune etiologies. Treatment is usually corticosteroids or immunosuppressive agents. Our patient did present with progressive dysphagia but had no other findings suggestive of proximal muscle weakness or cutaneous manifestations of DM. ESR, CRP, LDH, and AST, which are commonly elevated in DM and PM, were within normal limits, and CK levels were only mildly elevated. Furthermore, she was treated with steroids but did not demonstrate a significant clinical response. Diagnostic tissue biopsy did not reveal characteristic inflammatory cell infiltration, necrotic and regenerating muscle fibers, or atrophic muscle fibers that are characteristic of an inflammatory myositis. Although her CT scan was suspicious for myositis, her presentation and clinical course were not specifically suggestive of DM or PM.

The distinction between PM and the patient's myositis bears examination because PM is strongly associated with cancer, to such an extent that cancer surveillance and various imaging are warranted in new diagnoses [14]. Although less common, there are reported cases of paraneoplastic DM related to breast cancer [15, 16]. DM and PM are thought to manifest as paraneoplastic syndromes when cancer cells overexpress myositis-specific antigens (MSA) that cause autoimmune damage to both muscle and cancer mediated by T-cells [17]. However, in this case, myositis did not occur as a paraneoplastic syndrome mediated by T-cells; rather direct invasion of metastatic carcinoma cells caused the inflammatory changes seen on the patient's imaging and biopsy.

The nature of this breast cancer metastasis was also unusual. Skeletal muscle metastases are rare in and of themselves, likely due to a hostile microenvironment created by muscle's pH, ability to remove lactic acid associated with angiogenesis, the activation of lymphocytes and NK cells in skeletal muscles, and mechanical tumor destruction from motion. It is thought that for these reasons, skeletal muscle metastases from breast cancer are uncommon. However, this pattern of metastasis has been described and is usually seen with disseminated, multiorgan involvement [18]. When skeletal muscle metastases do develop, they often present as isolated, painful soft tissue masses in the involved areas [4, 18]. Typically, these metastases are challenging to evaluate with CT alone due to isodensity with surrounding muscle. They may be more appropriately identified with MRI or FDG PET-CT and are often found to have round or oval shapes with well-defined margins [19, 20]. However, in this case, the metastases manifested as diffuse infiltration of cervical musculature without a discrete lump or mass. Breast cancer presenting with direct muscle invasion in an infiltrative pattern mimicking myositis has only been reported in one other case [12]. Due to its relatively rare occurrence, there currently is no consensus on the standard treatment for skeletal muscle metastases, and further studies are needed to determine the prognosis and proper diagnostic and therapeutic treatment [18].

Given that imaging studies for this patient depicted what appeared to be impressive inflammatory stranding of the muscles of the neck, it is probable that subtler changes may have been detected earlier in the disease course prior to the development of respiratory failure. Earlier imaging in this case may have led to more timely intervention and prevention of this airway emergency. Various modalities to achieve airway patency are available. These options may include emergent tracheostomy, laser therapy, contact electrocautery, argon plasma coagulation, cryotherapy, photodynamic therapy, brachytherapy, and airway stenting [21].

## 4. Conclusion

This case illustrates a unique presentation of metastatic breast cancer presenting as a muscle infiltration mimicking myositis and resulted in airway compromise. Initial imaging showed distortion of tissue planes which appeared inflammatory in etiology thus confounding the differential diagnosis. This patient's initial subacute presentation of cough and dyspnea were nonspecific and would be unlikely to trigger a concern for breast cancer. However, close clinical follow-up and repeat imaging may have led to a more timely diagnosis thereby preventing the development of imminent respiratory failure. Earlier recognition may have allowed for the availability of wider array of palliative interventions to improve the patient's life expectancy and quality of life.

Unfortunately, this patient's clinical course was highly aggressive, and she ultimately required urgent intubation and subsequent tracheostomy. A high index of suspicion for neoplasm should be maintained when patients present with an indeterminate myositis. For patients with progressive dyspnea, dysphagia, and evidence of myositis by imaging, it is important to biopsy areas of involvement to evaluate for sarcoidosis, amyloidosis, drug-induced myositis, other infectious, and familial and autoimmune as well as neoplastic etiologies. A wide range of therapies are available to patients with airway compromise secondary to malignancy but should coincide with goals of care.

## References

[1] Iqbal J., Ginsburg O., Rochon P. A., Sun P., Narod S. A. (2015). Differences in breast cancer stage at diagnosis and cancer-specific survival by race and ethnicity in the United States. *Journal of the American Medical Association*.

[2] Leong S. P. L., Cady B., Jablons D. M. (2006). Clinical patterns of metastasis. *Cancer and Metastasis Reviews*.

[3] Kreisman H., Wolkove N., Finkelstein S., Cohen C., Margolese R., Frank H. (1983). Breast cancer and thoracic metastase: review of 119 patients. *Thorax*.

[4] Ogiya A., Takahashi K., Sato M. (2015). Metastatic breast carcinoma of the abdominal wall muscle: a case report. *Breast Cancer*.

[5] Surov A., Machens A., Holzhausen H.-J., Spielmann R. P., Dralle H. (2016). Radiological features of metastases to the thyroid. *Acta Radiologica*.

[6] Lacka K., Breborowicz D., Uliasz A., Teresiak M. (2012). Thyroid metastases from a breast cancer diagnosed by fine-needle aspiration biopsy. Case report and overview of the literature. *Experimental Oncology*.

[7] Rao M. Y., Wu J. B. (2015). Invasive ductal breast cancer with extensive subcutaneous metastases in trunk: a case report. *European Review for Medical and Pharmacological Sciences*.

[8] Agrawal S., Jayant K., Agarwal R. K. U., Dayama K. G., Arora S. (2015). An unusual case of metastatic male breast cancer to the nasopharynx-review of literature. *Annals of Palliative Medicine*.

[9] Tiwari V., Pande S. C. H., Verma K., Goel S. (2014). Paranasal sinus and retro-orbital metastasis in a case of breast carcinoma: a clinicoradiological review. *BMJ Case Reports*.

[10] Davey S., Baer S. (2012). A rare case of breast cancer metastasising to the nasopharynx and paranasal sinuses. *International Journal of Surgery Case Reports*.

[11] Bayon R., Banas S. K., Wenig B. L. (2013). Case report: Metastatic breast cancer presenting as a hypopharyngeal mass. *Ear, Nose and Throat Journal*.

[12] Noda S., Kashiwagi S., Kawajiri H., Takashima T., Onoda N., Hirakawa K. (2013). A case of metastatic breast carcinoma of the cervical muscles. *Gan To Kagaku Ryoho*.

[13] Murgu S. D., Egressy K., Laxmanan B., Doblare G., Ortiz-Comino R., Hogarth D. K. (2016). Central airway obstruction: benign strictures, tracheobronchomalacia, and malignancy-related obstruction. *Chest*.

[14] Fang Y.-F., Wu Y.-J. J., Kuo C.-F., Luo S.-F., Yu K.-H. (2016). Malignancy in dermatomyositis and polymyositis: analysis of 192 patients. *Clinical Rheumatology*.

[15] Sakai T., Ogura Y., Narita J. (2005). Metachronous bilateral primary breast cancer associated with dermatomyositis. *The Breast Journal*.

[16] Merali N., Yousuff M., Pronisceva V., Poddar A. (2017). Paraneoplastic polymyositis presenting as a clinically occult breast cancer. *The Annals of The Royal College of Surgeons of England*.

[17] Levine S. M. (2006). Cancer and myositis: new insights into an old association. *Current Opinion in Rheumatology*.

[18] Kim Y. W., Seo K. J., Lee S. L. (2013). Skeletal muscle metastases from breast cancer: two case reports. *Journal of Breast Cancer*.

[19] Emmering J., Vogel W. V., Stokkel M. P. M. (2012). Intramuscular metastases on FDG PET-CT: a review of the literature. *Nuclear Medicine Communications*.

[20] Bello-Roufai D., Soares D. G., Kerrou K. (2017). Long-term complete response in a breast cancer patient with skeletal muscle metastases diagnosed using 18F-FDG-PET. *Oxford Medical Case Reports*.

[21] Breitenbücher A., Chhajed P. N., Brutsche M. H., Mordasini C., Schilter D., Tamm M. (2008). Long-term follow-up and survival after Ultraflex stent insertion in the management of complex malignant airway stenoses. *Respiration*.

